# Fault Detection of Wind Turbine Gearboxes Based on IBOA-ERF

**DOI:** 10.3390/s22186826

**Published:** 2022-09-09

**Authors:** Mingzhu Tang, Chenhuan Cao, Huawei Wu, Hongqiu Zhu, Jun Tang, Zhonghui Peng, Yifan Wang

**Affiliations:** 1 School of Energy and Power Engineering, Changsha University of Science & Technology, Changsha 410114, China; 2 Hubei Key Laboratory of Power System Design and Test for Electrical Vehicle, Hubei University of Arts and Science, Xiangyang 441053, China; 3 School of Automation, Central South University, Changsha 410083, China

**Keywords:** fault detection, butterfly optimization algorithm, extreme random forest, wind turbine, gearbox

## Abstract

As one of the key components of wind turbines, gearboxes are under complex alternating loads for a long time, and the safety and reliability of the whole machine are often affected by the failure of internal gears and bearings. Aiming at the difficulty of optimizing the parameters of wind turbine gearbox fault detection models based on extreme random forest, a fault detection model with extreme random forest optimized by the improved butterfly optimization algorithm (IBOA-ERF) is proposed. The algebraic sum of the false alarm rate and the missing alarm rate of the fault detection model is constructed as the fitness function, and the initial position and position update strategy of the individual are improved. A chaotic mapping strategy is introduced to replace the original population initialization method to enhance the randomness of the initial population distribution. An adaptive inertia weight factor is proposed, combined with the landmark operator of the pigeon swarm optimization algorithm to update the population position iteration equation to speed up the convergence speed and improve the diversity and robustness of the butterfly optimization algorithm. The dynamic switching method of local and global search stages is adopted to achieve dynamic balance between global exploration and local search, and to avoid falling into local optima. The ERF fault detection model is trained, and the improved butterfly optimization algorithm is used to obtain optimal parameters to achieve fast response of the proposed model with good robustness and generalization under high-dimensional data. The experimental results show that, compared with other optimization algorithms, the proposed fault detection method of wind turbine gearboxes has a lower false alarm rate and missing alarm rate.

## 1. Introduction

As an important source of clean and renewable energy, wind energy resources play an important role in the sustainable development of the national economy. The use of wind power is very environmentally friendly, and wind energy reserves are huge, so wind power is attracting more and more attention from countries all over the world. According to the forecast of the Global Wind Energy Council (GWEC), global wind power will increase by 557 GW in the next five years (2022–2026), with a compound annual growth rate of 6.6%. By 2026, the global newly installed capacity of wind power will reach 128.8 GW, of which the newly installed capacity of onshore wind power will be 97.4 GW, while the newly installed capacity of offshore wind power will be 31.4 GW [1]. However, abundant wind resources are often found in remote areas, and the occurrence of some extreme weather conditions can lead to the failure of wind turbines [2]. Compared with the tower base, the narrow nacelle does not have a solid foundation, and the factors of power matching and torsional deformation in the drive train are always concentrated in a weak link. Much research has proven that this link is often the gearbox in the unit [3]. The gearbox is an essential mechanical component, and its main purpose is to transport the power generated by the blades to the power generator in order to obtain the appropriate speed [4]. Due to its special installation position, once a fault occurs, it is very difficult to repair. Compared with other unit components, the gearbox has the longest downtime and repair time due to failure, resulting in long-term gearbox downtime. Therefore, providing accurate guidance at the first instance of failure can reduce the operating cost and maintenance cost of the wind turbine, which has great economic and engineering value [5]. In recent years, scholars have carried out extensive applied research on the fault detection of wind turbines.

Currently, research on fault detection of wind turbine gearboxes mainly includes methods based on signal processing, along with data-driven and model-based methods [6,7,8]. Signal-based approaches—such as spectral analysis, wavelet transform [9], and non-parametric spectrum estimation—are often carried out. However, for stationary signal power, unlike the theoretical infinite-length signal, the actual observed signal is a finite-length signal. Low resolution of frequency is inevitable in the conversion process. The data-driven approach requires large volumes of historical data and multidimensional features [10]. Today, machine-learning-based fault detection approaches are used extensively in the field of industry [11].

In machine learning, the decision tree classification model is a tree structure, which is strongly intuitive and easy to understand, and has become a popular technology of online detection. Liang [12] proposed to encrypt the decision table using a searchable symmetric encryption method to improve the classification speed and solve the detection requirement in microseconds. Stetco [13] reviewed the machine learning methods used in wind turbine blades, generator temperature fault detection, etc. Classification is mostly used when using SCADA datasets or simulation data, and decision trees are the most commonly used models. In general, decision trees are prone to overfitting and poor generalization performance, and small changes in the data may lead to the generation of completely different trees—that is, their stability performance needs to be improved. To solve this problem, Feng [14] used the adaptive boost algorithm to find the mapping between incoming data and outgoing data, and the overall accuracy of the model was improved.

The boost algorithm in machine learning refers to integrating multiple weak classifiers to reduce the time complexity of a single decision tree and make the model easy to display [15]. Liu [16] proposed a fault detection method based on NFSW-BP-AdaBoost to evaluate the combination of multiple classifiers with non-fuzzy solution coefficients to improve the recognition rate of faults. Chakraborty [17] designed the data-driven model of extreme gradient boosting (XGBoost), using the dynamic adjusted threshold to judge the occurrence of faults, which improved the quality of the model and had strong generalization ability. Xu [18] designed cost-sensitive GBDT (CS-GBDT) to improve the problem of low diagnostic accuracy in the face of unbalanced datasets, and used multiple-domain feature extraction and feature selection to enhance diagnostic accuracy. However, in the face of high-dimensional complex data in actual wind farms, the boost algorithm consumes too much memory, making it easy to reduce the calculation accuracy and fault detection accuracy.

Owing to the large amount of data and high dimensionality of real wind farms, existing studies usually have problems such as poor performance and long training time. Extreme random forest is an ensemble tree algorithm with complete randomness proposed on the basis of decision trees. The feature values are selected for segmentation in the training phase to obtain the segmentation values. This method has strong randomness, and in practical applications it shows high accuracy in high-dimensional datasets, can easily achieve parallelization, and has strong generalization performance. However, in the domain of practical fault detection, the selection of hyperparameters is extremely critical to the final detection results, and suitable hyperparameters can prevent the local convergence of the model and achieve the best results [19].

For high-dimensional nonlinear problems, the modern intelligent optimization algorithm is widely used in the field of fault detection [20]. In practical applications, the optimization algorithm is used to find the optimal scheme or parameter value among many schemes or parameter values, so that some performance and function indices of the system can reach optimal values. Arora [21] introduced a new nature-inspired heuristic algorithm—the butterfly optimization algorithm, which has the strengths of requiring few adjustment parameters and strong convergence. However, in the face of complex optimization problems such as high-dimensional data, it is prone to being trapped in local optima, and another problem is its slow convergence speed [22].

In view of the above problems, a fault detection model with extreme random forest optimized by improved butterfly optimization algorithm (IBOA-ERF) was proposed. In the improved butterfly optimization algorithm, chaotic mapping is introduced to initialize the population, and the adaptive inertia weight factor is introduced. Combined with the pigeon swarm optimization algorithm, adaptive dynamic switching is proposed to control the conversion of the search stage, which is integrated into the population position update formula, and the convergence speed and optimization accuracy are greatly improved. Firstly, the data are cleaned using Pearson’s correlation analysis, reducing the data’s dimensions and deleting redundant features. Secondly, the sample dataset is divided into two categories: a training set and a test set. The improved butterfly algorithm is used to generate the best hyperparameters of the extreme random forest, and the IBOA-ERF fault detection model is constructed to detect the gearbox faults of wind turbines.

## 2. Fault Detection of Wind Turbine Gearboxes

As one of the most significant structural parts of a wind turbine, the gearbox is subject to very complex forces, and works under complex alternating loads and harsh working environments for a long time.

**Figure 1:**
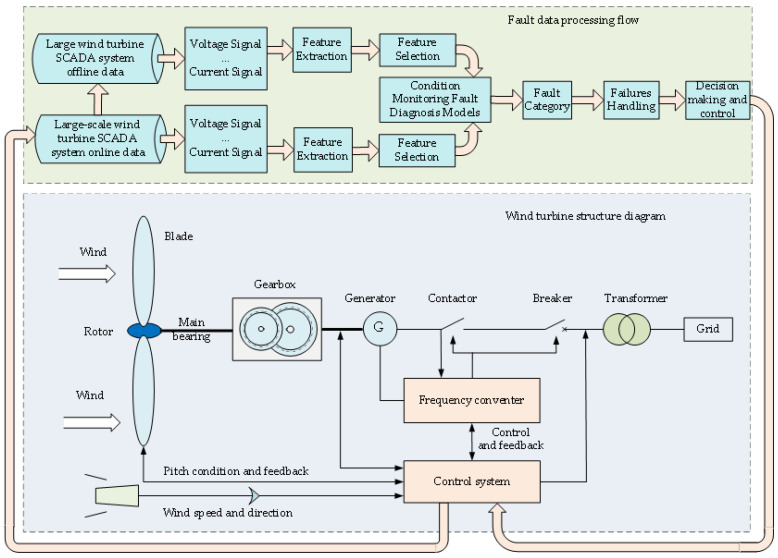
Schematic diagrams of a wind turbine’s structure and the fault detection process.

Figure 1 shows schematic diagrams of a wind turbine’s structure and the fault detection process. When the unsteady wind acts on the unit, different loads are generated [23]. The blade produces axial thrust and circumferential shear, resulting in deflection movement [24]. The torsional main bearing transmits the blade torque to the gearbox to complete the output of the corresponding load. In the generator, the torque on the motor shaft continuously cuts the magnetic induction line to output power, and completes the conversion of wind energy, mechanical energy, and power [25]. Subsequently, the coordination of major electrical parameters and data interaction is completed through the frequency converter and control unit. The actual operating data of the wind turbine are stored in the SCADA system, making it easy to extract data for fault detection.

The proportion of failures caused by broken teeth, pitting, gluing, and wear of gears inside the gearbox is about 60%, while the proportion of failures caused by damage to bearings such as burns, balls falling off, and cage deformation is about 20%, which seriously impact the security and stability of the whole machine’s operation [26]. Due to the high fault dimensions and redundant parameters, it is important to mine the fault characteristics of gearboxes deeply and determine the fault location and category quickly and accurately for the secure and stable operation of wind turbines.

In summary, in order to further enhance the stability and precision of wind turbine gearbox fault detection, aiming at the problems of gearbox fault data dimension reduction, feature selection, and model parameter optimization, combined with extreme random forest with excellent classification performance, a wind turbine fault detection model based on IBOA-ERF is adopted, which improves the detection precision of the model and ensures the safe operation of the wind turbine.

## 3. Extreme Random Forest

**Figure 2:**
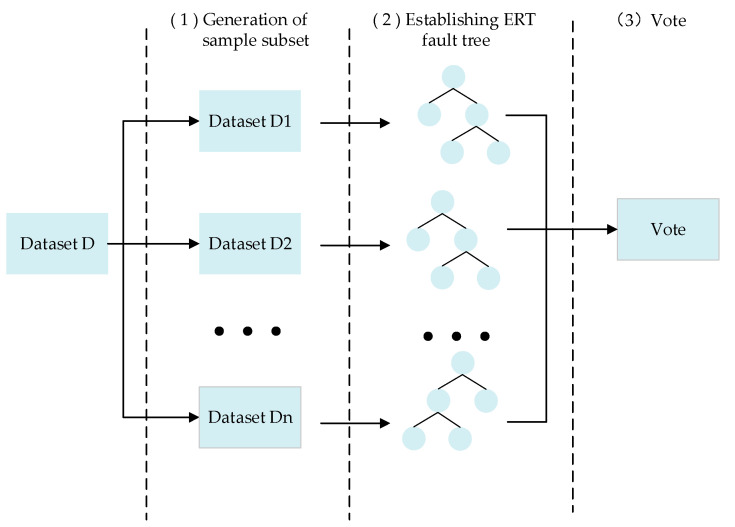
Structure diagram of ERF.

**Figure 3:**
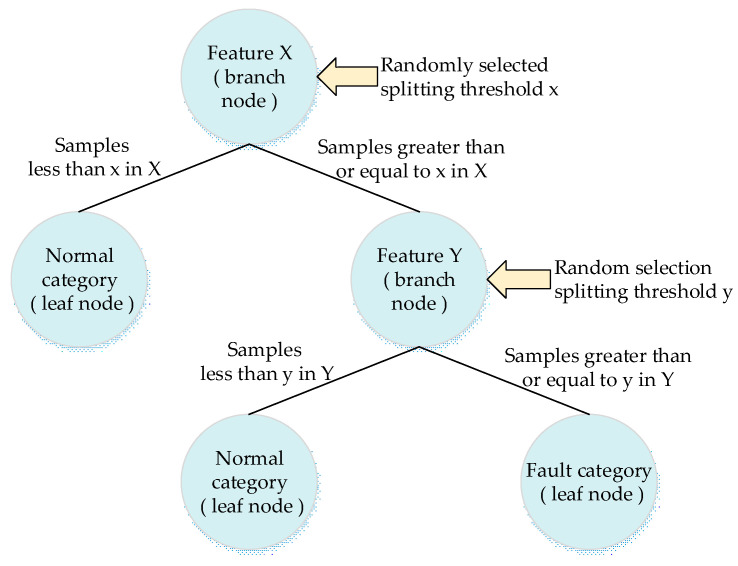
Illustration of the splitting architecture of the ERF fault tree.

Random forest (RF) consists of a series of decision trees. The decision tree is a tree structure, in which each internal node represents a categorical judgment, and each leaf node at the bottom represents a classification result; this is detailed in Figure 2 and Figure 3. A subset of n samples of the same size as the sample set is obtained by randomly selecting the sample set. Next, several weak classifiers are built. A decision tree is a tree classification method derived from the training samples by using a set of random vectors.

At the time of node-splitting, through top-down recursion, traversing each feature and each value of each feature, and use evaluation criteria such as the Gini coefficient to determine the optimal features and feature values as node features and thresholds. The process iteratively splits down until the entropy of each leaf node is reduced to 0—that is, the class confusion degree of the sample is 0—and then votes to determine the final classification. Through the above steps, the unique path of each sample is determined, and the category of the sample is the category corresponding to the leaf node of the unique path.

While inheriting the good performance of RF, extreme random forest (ERF) has two main differences: First, the original dataset is used in the training set of each decision tree. Due to the randomness of feature selection and node splitting, the obtained results will be better than those of RF. Second, after picking the segmentation features, RF selects an optimal feature value for segmentation, while the ERF splits the randomly selected eigenvalues, which enhances the generic performance of the model, while the size of the decision tree increases. Figure 2 shows a structural diagram of ERF.

The class attribute is determined by the vote of all decision trees, and its vote is based on Equation (1). The larger the calculated P, the higher the probability of belonging to the corresponding category. Equation (2) is the voting mechanism principle of the final decision tree. The above method is used to generate the extreme random forest decision tree.

(1) $$P(c|f_{i})=\frac{1}{D}\overset{D}{\underset{t=1}{\sum }}P_{t}(c|V_{i})$$

(2) $$\hat{c}={\mathrm{argmax}}_{c}\;P(c|V_{i})$$

where $V_{i}$ denotes the feature vector of the sample, c is some kind of category, D denotes the number of trees in the ERF, $P_{t}(c|V_{i})$ denotes the probability that the sample belongs to category c conditional on the feature vector $V_{i}$, $P(c|V_{i})$ is the average value in the ERF, and $\hat{c}$ represents the category corresponding to the maximum value of $P(c|V_{i})$.

During the node-splitting phase, for the process of selecting the obtained feature as the splitting feature, Equation (3) is used to measure the score. When the leaf nodes are split, the splitting feature is selected as the feature with the highest score. Samples smaller than the splitting threshold are put in the left leaf node after splitting; otherwise, they are placed in the right leaf node. These procedures are repeated until the sample confusion in the leaf node is 0. Figure 3 illustrates the splitting architecture of the ERF fault tree.

(3) $${\mathrm{Score}}_{k}=\frac{2I_{k}}{H_{k}+H_{c}}$$

where ${\mathrm{Score}}_{k}$ represents the score measurement of the calculated feature, and $I_{k}$ denotes the mutual information of the two subsets of the node after splitting on the basis of the corresponding features and splitting threshold of the sample category. $H_{k}$ denotes the split entropy of feature k, while $H_{c}$ represents the information entropy of the node for the corresponding category.

The choice of hyperparameters in ERF has a great influence on the classification precision of the model, and the optimization of the parameters is difficult. Therefore, optimization algorithms must be introduced to search for the best parameters to enhance the reliability of the fault detection model.

## 4. Butterfly Optimization Algorithm

In nature, butterflies use their high sensitivity to fragrance to search for food and partners. In 2019, Arora [21] proposed the butterfly optimization algorithm (BOA), which imitates the movements of butterflies in search of food and mating.

### 4.1. Basic Theory of the Butterfly Optimization Algorithm

Studies have shown that butterflies can accurately determine the location of food by detecting different flavors and flavor intensity during predation [27]. In the butterfly optimization algorithm, each butterfly produces a certain intensity of fragrance according to its fitness, and when it perceives that the fragrance emitted by another butterfly in a certain region is stronger, it will try to approach this butterfly, which is known as global search. When a butterfly perceives its own fragrance to be more intense than that of other butterflies, it will be able to freely move in space, which is known as local search [28].

In the BOA, butterfly fragrance calculation is as shown in Equation (4):

(4) $$f={\mathrm{sI}}^{\alpha }$$

where f is the fragrance intensity, I is the stimulus intensity, s is the sensory modality with a value of 0.01, and α is the power exponent with a value of 0.1.

In the BOA, the stimulus intensity I of the individual is influenced by the objective function, and the power exponent α is the exponent of the increase in fragrance intensity. The transitions of the global and local search stages are controlled by the switching transition frequency p ∈ [0, 1]. In the global search phase, the position is updated as shown in Equation (5):

(5) $$x_{i}^{t+1}=x_{i}^{t}+\left(r^{2}\times g^{*}-x_{i}^{t}\right)\times f_{i}$$

where $x_{i}^{t+1}$ and $x_{i}^{t}$ are the location information of the i-th individual in the t+1-th and t-th iterations, respectively; $g^{*}$ is the best value in the current iteration; $f_{i}$ is the fragrance intensity emitted by the i-th individual; and r is the random value from 0 to 1. In the local search phase, the position is updated as shown in Equation (6):

(6) $$x_{i}^{t+1}=x_{i}^{t}+\left(r^{2}\times x_{j}^{t}-x_{k}^{t}\right)\times f_{i}$$

where j and k are the random numbers generated in each iteration, while $x_{j}^{t}$ and $x_{k}^{t}$ are the location information of the j-th and k-th individuals in the current iteration, respectively.

### 4.2. Improvement and Innovation of the Butterfly Optimization Algorithm

Compared with some existing meta-heuristic algorithms, the BOA is relatively novel, with simple operation, few parameters to be adjusted, and better robustness. It is superior to some classic intelligent optimization algorithms in terms of optimization ability, and has achieved good results in the preliminary application of engineering practice. However, in the face of complex conditions, its performance is not good, and there are still problems such as its tendency to become trapped in local optima and its low convergence precision when solving high-dimensional functions. To solve this problem, the improved butterfly optimization algorithm (IBOA) is constructed through the following four modifications:

1. Introduce a chaotic map to randomly initialize the population position, so that the initial population is random and aperiodic, so as to prevent the exploration process from ending up in a local optimum.
2. Design an adaptive inertia weight factor and apply it to the position update formula to enhance the capability of local search and accelerate the search rate.
3. Introduce the landmark operator sub-item of the pigeon group optimization algorithm, design a new position update formula, enhance the global search capability, and improve the diversity and robustness of the butterfly optimization algorithm.
4. Design a new dynamic switching method for the local search phase and the global search phase, and introduce the variant of trigonometric function as the switching basis, which can effectively prevent trapping in local optima and accelerate the convergence speed.

#### 4.2.1. Chaos Map Initialization

**Figure 4:**
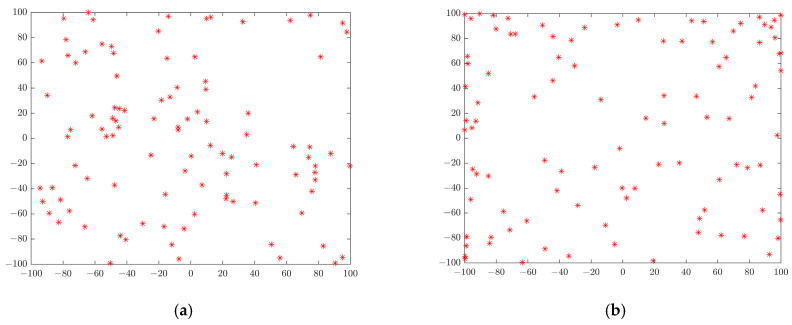
(**a**) The distribution after random initialization; (**b**) the distribution after initialization of the chaotic map.

BOA randomly initializes the population position, but using this approach to generate the initial population may lead to uneven distribution and superposition of individual butterfly positions. In the butterfly population, the small change in the initial distribution has a great impact on the subsequent iterative search process. To solve this problem, chaotic variables are used to optimize the search so as to evenly distribute the initial population [29], which can improve the diversity of BOA, greatly improve the convergence speed and optimization accuracy, and prevent premature convergence. After testing and comparison, the classical logistical chaotic mapping is used to initialize the population. The logistic map described in [30] is used to map the variables into the chaotic variable space, and then used the linear transformation to map the generated chaotic variables into the solution space in need of optimization. Figure 4 shows the comparison between the initialization using chaotic mapping and the original initialization method. The specific expression of the logistic map is as shown in Equation (7):

(7) X(t+1)=X(t)×μ×(1−X(t)) μ∈[0,4], X∈[0,1] 

where μ is the logistics parameter, X is the position parameter, and t is the value of the iterations. The research shows that when μ is 4, the range of X is almost evenly distributed in the entire region of 0 to 1, so the value of μ in this case is 4.

#### 4.2.2. Adaptive Inertia Weighting Factor

According to the basic principle of the BOA, each individual updates or randomly moves its position according to the current best individual position. Therefore, the position of individual butterflies is not fully utilized, and it is easy to become trapped in a local optimum. When the inertia factor is large, the global search capability is strong, and vice versa. Therefore, to address this issue, an adaptive inertia weighting factor was designed to apply to the position update formula, so that the historical optimal position information of the individual is fully utilized. Meanwhile, as the iterations grow in size, the direction and distance of the individual are effectively controlled, so as to enhance the optimization precision and convergence velocity, and avoid falling into local optima. The expression of the inertia weighting factor is as follows:

(8) $$\omega \left(t\right)=1-\sin\left(\frac{\pi t}{\sqrt{e+1}\times T_{\mathrm{iter}}}\right)$$

where ω is the adaptive inertia weight, $T_{\mathrm{iter}}$ is the largest value of the number of iterations t in the optimization process, and e is the Euler number.

The position update formula for the global search phase after the introduction of the adaptive inertia weighting factor in BOA is as follows:

(9) $$x_{i}^{t+1}=\omega \left(t\right)\times x_{i}^{t}+\left(r^{2}\times g^{*}-x_{i}^{t}\right)\times f_{i}$$

The position update formula for the local search phase is as follows:

(10) $$x_{i}^{t+1}=\omega \left(t\right)\times x_{i}^{t}+\left(r^{2}\times x_{j}^{t}-x_{k}^{t}\right)\times f_{i}$$

#### 4.2.3. Pigeon-Inspired Optimization Algorithm Landmark Operator

Inspired by the nesting activity of pigeons, a new population intelligence optimization algorithm—the pigeon-inspired optimization (PIO) algorithm—was first proposed by Duan [31] in 2014.

PIO simulates pigeon homing using different search mechanisms at different stages. The algorithm includes two models: a compass model and a landmark model. In the compass model, the individual updates the location according to its previous location information and the current global optimal location information. In the landmark operator, on the basis of halving the number of groups in each iteration, the pigeons accelerate the convergence rate according to the average value of group fitness. PIO has the characteristics of fast convergence and high search accuracy, and has been widely used in different fields [32].

The landmark model of PIO is as follows:

(11) $$x_{i}^{t+1}=x_{i}^{t}+r\times \left(x_{c}^{t}-x_{i}^{t}\right)$$

(12) $$x_{c}^{t}=\frac{\sum^{\;}\left(x_{i}^{t}\times \mathrm{Fit}\left(x_{i}^{t}\right)\right)}{N_{p}^{t}\times \sum^{\;}\mathrm{Fit}\left(x_{i}^{t}\right)}$$

(13) $$N_{p}^{t+1}=\frac{N_{p}^{t}}{2}$$

where $x_{c}^{t}$ is the position of the center of the flock in the current iteration, $\mathrm{Fit}\left(x_{i}^{t}\right)$ is the value of the fitness function of the i-th pigeon, and $N_{p}^{t}$ is the number of individuals. Other variables are defined as in Equation (5).

In the BOA, the fragrance of butterflies plays an important role in guiding individuals to move to the optimal solution. However, if the population falls into the local optimal position, it is prone to resulting in a stagnant search that does not lead to a globally optimal resolution. Based on this problem, inspired by PIO, combined with the landmark model, a new butterfly position update formula was constructed. Since the landmark model needs to calculate the average fitness of the group, compared with the compass model, not only is the global search capability greatly enhanced, but also the convergence velocity is improved. The improved butterfly position global search stage update formula is as follows:

(14) $$x_{i}^{t+1}=\omega \left(t\right)\times x_{i}^{t}+\left(r^{2}\times x_{j}^{t}-x_{k}^{t}\right)\times f_{i}+r\times \left(x_{c}^{t}-x_{i}^{t}\right)$$

#### 4.2.4. Adaptive Dynamic Switching

**Figure 5:**
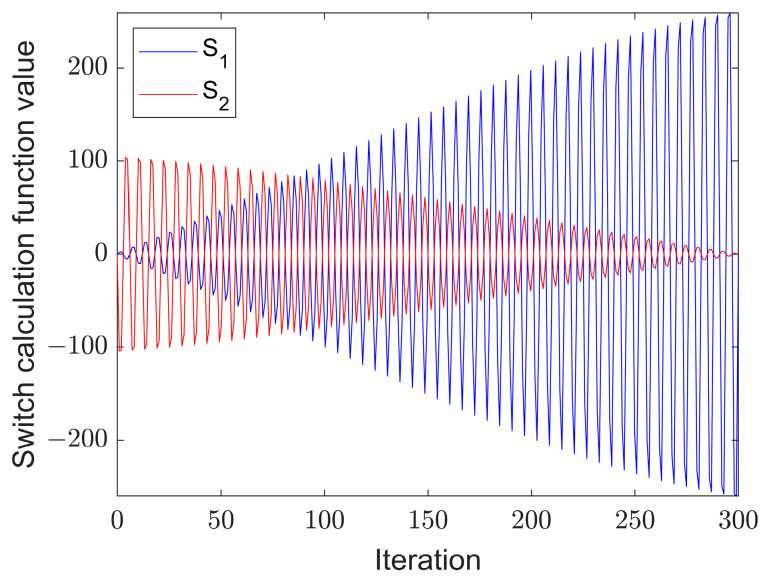
Switching between global and local search phases.

In the BOA, the switching between the local search stage and the global search stage is controlled by the switching frequency p. The higher the value of the parameter p, the greater the proportion of global search; the lower the value of p, the greater the proportion of the local search. The value of p plays a key role in the subsequent search efficiency and convergence rate. To solve this problem, an adaptive dynamic switching frequency strategy is proposed. The oscillation trigonometric function is introduced. The proportion of local and global search stages is dynamically adjusted according to the number of iterations. The random selection search phase is changed in such a way that global search is performed in the early stage, while local search is performed in the middle and late stages.

(15) $$S_{1}\left(t\right)=\left(t+1\right)\times \sin\left(\mathrm{wt}\right)$$

(16) $$S_{2}\left(t\right)=\sqrt{e-\emptyset }\times ((T_{\mathrm{iter}}-t)+1)\times \sin\left(w\times \left(T_{\mathrm{iter}}-t\right)\right)$$

where w and ∅ take the values 100*π and 2.55, respectively, while e is the Euler number. The iterative process, as shown in Figure 5, enters the local search phase when |S_1_(t)| > |S_2_(t)|, and otherwise enters the global search phase, which can be experimentally proven to converge faster and search more efficiently.

### 4.3. Simulation Experiments

In order to verify that the IBOA has better performance in terms of convergence and robustness, a performance comparison experiment was carried out based on six test functions: F1~F3 are unimodal functions to test the convergence performance of the algorithm, while F4~F6 are complex multimodal functions to test global optimization and jump out of local optimization performance. The standard test function information is shown in Table 1.

In order to sufficiently validate the effectiveness of the IBOA, the comparative experiments were conducted with moth–flame optimization (MFO) [33], multi-verse optimization (MVO) [34], the sine–cosine algorithm (SCA) [35], the salp swarm algorithm (SSA) [36], and the BOA. The number of iterations was 500, and each method was run 30 times separately on each test function to prevent bias in the outcomes due to random factors, as detailed in Table 2.

**Figure 6:**
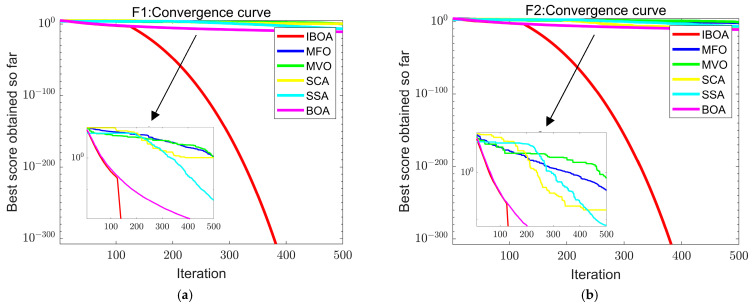

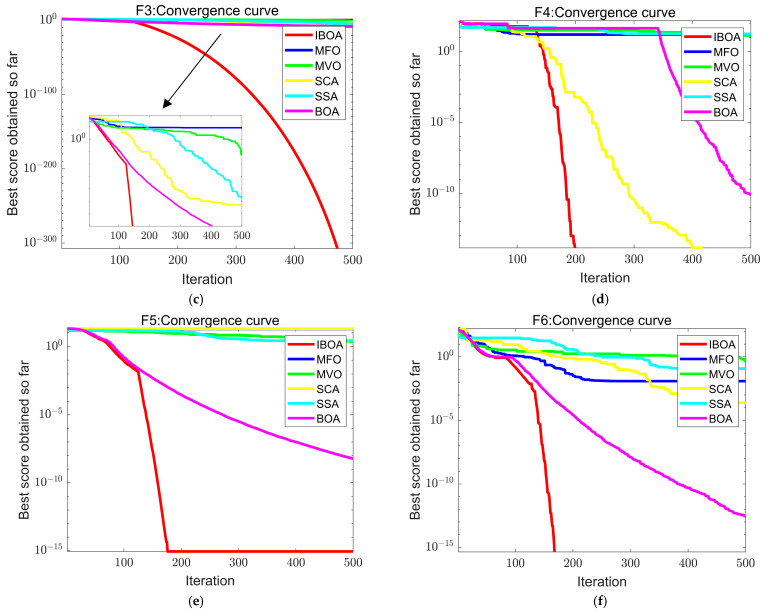
(**a**) Convergence curve of function F1; (**b**) convergence curve of function F2; (**c**) convergence curve of function F3; (**d**) convergence curve of function F4; (**e**) convergence curve of function F5; (**f**) convergence curve of function F6.

To visually demonstrate the optimized capabilities of the IBOA, the iterative graph of the convergence curve of the six benchmark functions was selected, as shown in Figure 6.

### 4.4. Analysis of Simulation Experiment Results

When solving the minimum value problem, the average value is used to evaluate the optimal ability and convergence precision, the standard deviation is used to evaluate the robustness, and the best value and the worst value are used to evaluate the quality of the feasible solution of the algorithm.

As shown in Table 2, in terms of optimal values, the IBOA does not significantly improve in the F5 function, but it still has a great progress trend compared with the basic BOA, and the optimal value is found in other functions, indicating that the initialization of the population position through chaotic mapping maintains the diversity of the algorithm.

From an average perspective, the IBOA’s performance is far superior to that of other algorithms, especially in the unimodal function, indicating that the new location update equation combined with the pigeon swarm algorithm and the strategy of dynamic search-stage switching not only accelerates the convergence speed, but also further enhances the quality of the refined search at a later stage, and greatly improves the overall optimization ability.

From the perspective of standard deviation, the capability of the IBOA is significantly superior to that of other methods; the optimization ability is significantly enhanced, and the quality of the IBOA’s feasible solutions is high, indicating that the introduction of the adaptive inertia weighting factor strategy in the position update equation effectively maintains the population diversity, improves the global optimization ability, and maintains strong robustness throughout the search process, so as to acquire the global optimal solution.

## 5. ERF Fault Detection Model Based on the IBOA

### 5.1. Data Pre-Processing

**Figure 7:**
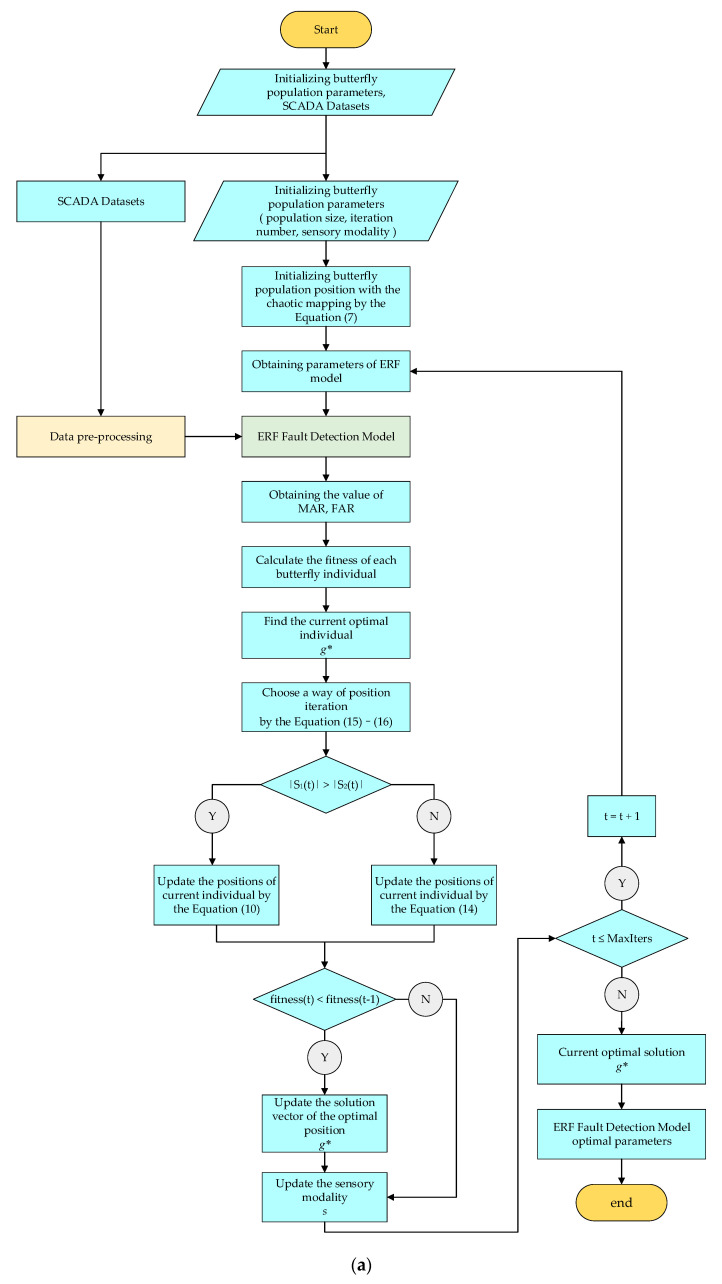

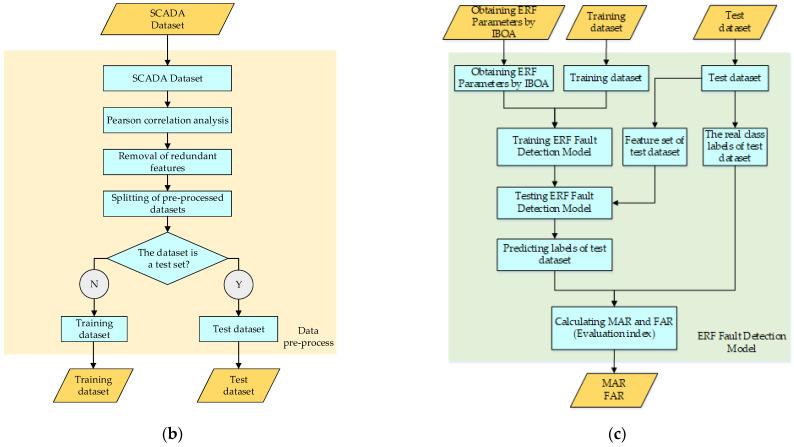
(**a**) The flow chart of IBOA to find the optimal parameters; (**b**) the flow chart of Data pre-process; (**c**) the flow chart of ERF Fault Detection Model.

The operation process of the wind turbine gearbox is complex, the state quantity generated is complex, and there are many redundant variables, increasing the complexity of model training and affecting the prediction performance of the model [37]. As illustrated in Figure 7b, it is important that the data gathered from the SCADA dataset undergo preliminary data cleaning, and then Pearson’s correlation analysis is performed to remove redundant feature values [38]. Pearson’s correlation coefficient is illustrated in Equation (17):

(17) $$\rho_{X,Y}=\frac{\mathrm{cov}\left(X,Y\right)}{\sigma_{X}\times \sigma_{Y}}$$

where ρ represents the correlation coefficient between features in the sample, σ represents the standard deviation of the corresponding features, and cov represents the covariance between features.

Pearson’s correlation coefficient is the upgrade of Euclidean distance, and provides standard data input for the wind turbine gearbox fault detection model. Through Pearson’s correlation analysis, redundant features with low partial correlation are removed, making the model training more efficient and the prediction results more accurate [39].

### 5.2. ERF Fault Detection Model Flowchart and Pseudocode Based on the IBOA

After Pearson’s correlation analysis, the dataset is divided into two categories: the training dataset is utilized to train the classification model, while the test dataset is utilized for the prediction of the model, measuring the performance and classification ability of the model, and evaluating the model’s prediction performance.

The optimization of the IBOA parameters is shown in Figure 7. Firstly, the position and sensory mode of each individual are initialized to obtain the best adaptive value of the group. According to the adaptive dynamic switching, the local search or global search is selected. The corresponding position’s iterative formula is used to update the individual position, and the ERF model parameters are output to meet the iterative conditions. After obtaining the ERF model parameters, the ERF fault detection model based on the IBOA (IBOA-ERF) is constructed with the training data. The performance of the test model is tested by the real class labels of the test dataset and the predicted class labels generated by the model.

Table 3 shows the optimized ERF hyperparameters τ and δ in the IBOA model, including the meanings and ranges of the parameters.

Algorithm 1 is the pseudo-code of the IBOA model’s parameters. Algorithm 2 is the pseudo-code of ERF fault detection model using optimal parameters. The detailed optimization process of the model hyperparameters is as follows:
**Algorithm 1.** The steps of IBOA optimization parameters**Input:** IBOA parameters (*lb*(*τ_min_*, *δ_min_*), *ub*(*τ_max_*, δ*_max_*); dimension: *dim*; maximum number of iterations: *MaxIter*; population size: *N*; ERF parameters (τ, δ*)*;**Output:**g* (*τ_optimal_*, *δ_optimal_*);1:x_train, y_train, x_test, y_test → ERF (τ,δ)2:Initialize the butterfly population *N* (i = 1, 2, …, *N*)3:Calculate the fitness of each butterfly4:g* (τ,δ) = the best individual5:Build the fitness function: *fitness = FAR +*
ε ∗ *MAR*6:**While** t < *MaxIter*7:        **for** i = 1: *N*8:                Calculate the perceived magnitude of the fragrance using Equation (4)9:        **end for**
10:        Find the optimal butterfly individual g*
11:        **for** i = 1: *N*12:                **if** $\left|S_{1}\left(t\right)\right|>|S_{2}\left(t\right)|$13:                       Enter the local search phase based on Equation (10)14:                **else**
15:                       Enter the global search phase based on Equation (14)16:                **end if**
17:        **end for**
18:        Check if each butterfly exceeds the search space and correct for it19:Calculate the fitness of each butterfly20:Select the location that matches the minimum fitness value21:Update the value of α
22:        If a better solution is available, update g*23:        t = t + 1
24:**end while**25:**return**g* (*τ_optimal_*, *δ_optimal_*)

**Algorithm 2.** ERF Fault Detection Model**Input:** the best parameter vector g* (*τ_optimal_*, *δ_optimal_*); Training dataset; Test dataset;**Output:** MAR, FAR1:     Training dataset → x_train, y_train2:     Test dataset → x_test, y_test3:     x_train, y_train → ERF (*τ_optimal_*, *δ_optimal_*)4:     Training ERF fault detection model using Training dataset5:     x_test, y_test → ERF (*τ_optimal_*, *δ_optimal_*)6:     Testing ERF fault detection model using Test dataset7:     Obtaining predicted labels of test datasets8:     Calculating MAR and FAR of model performance based on Equations (18) and (19)9:     **return** MAR, FAR

## 6. Experimental Analysis

### 6.1. Dataset Description

To validate the validation of the proposed IBOA-ERF fault detection model, the annual gearbox operation data were extracted from the SCADA dataset with an interval of 1 min for a 1.5 MW wind turbine in China, and the data structure was selected from 30 min before the occurrence of the gearbox fault to 30 min after the end of the fault through the analysis of the wind turbine structure, as shown in Table 4.

For the purposes of the dataset, as illustrated in Table 5, the dataset can be divided into two parts: Dataset 1, with data on gearbox supercapacitor overtemperature faults and fault-free data; and Dataset 2, with data on gearbox nacelle operation overspeed faults and fault-free data.

### 6.2. Criteria for Evaluation

For the dichotomous problem of wind turbine gearbox fault detection, a confusion matrix was introduced. As illustrated in Table 6, the missing alarm rate (MAR) and the false alarm rate (FAR) of the matrix were utilized as evaluation indices.

(18) $$\mathrm{MAR}=\frac{S_{\mathrm{FN}}}{S_{\mathrm{FN}}+S_{\mathrm{TP}}}$$

(19) $$\mathrm{FAR}=\frac{S_{\mathrm{FP}}}{S_{\mathrm{FP}}+S_{\mathrm{TN}}}$$

where $S_{\mathrm{FN}}$, $S_{\mathrm{FP}}$, $S_{\mathrm{TN}}$, and $S_{\mathrm{TP}}$ represent the corresponding sample size.

To validate the excellence of ERF under the IBOA for the above extracted dataset, after data pre-processing, it was compared with the ERF model under MFO, MVO, SSA, SCA, and BOA optimization, and evaluated the performance of each model using MAR and FAR. Lower values of MAR and FAR represent better performance of the model. In order to prevent overfitting and improve model accuracy, each model was trained using 10-level cross-validation when conducting the comparison experiments. At the same population size and number of iterations, each model was run 10 times individually.

### 6.3. Experimental Results

When comparing the MAR and FAR of the ERF model under different optimization algorithms, IBOA-ERF performed better than the other five models.

**Figure 8:**
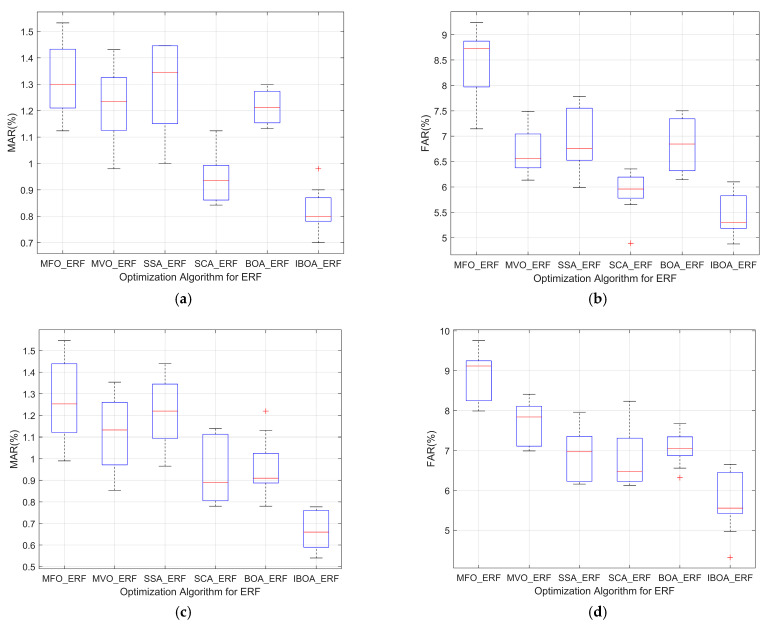
(**a**) MAR of six algorithms for fault detection on Dataset 1; (**b**) FAR of six algorithms for fault detection on Dataset 1; (**c**) MAR of six algorithms for fault detection on Dataset 2; (**d**) FAR of six algorithms for fault detection on Dataset 2.

For Dataset 1, as shown in Figure 8a, for the MAR of the six models, the average MAR of IBOA-ERF running 10 times alone was 0.86%, which is significantly improved compared with the BOA algorithm, and the fault detection ability is very stable. The overall MAR was maintained at 0.72–0.98%, while that of the other models was maintained at 0.84–1.53%. The optimization ability and optimization accuracy of the model were greatly improved. As shown in Figure 8b, for the FAR of the six models, the average FAR of IBOA-ERF running alone 10 times was 5.30%. During the detection process, the FAR of MFO-ERF was up to 9.23%, and the optimization effect was not obvious, while that of IBOA-ERF was maintained between 4.87% and 5.91%, and the detection performance was very stable. This shows that the ERF model has lower MAR and FAR, and the convergence efficiency and optimization performance are greatly improved when using the optimization parameters of the IBOA.

For Dataset 2, as shown in Figure 8c, the MAR of the ERF model under the IBOA had a maximum decrease of 1.06% compared to the other five models, showing less fluctuation than the classification results of the other models—which were generally maintained between 0.54% and 0.77%—along with significantly improved detection performance compared to the other models. As shown in Figure 8d, the FAR of the ERF model under the IBOA was generally stable between 4.97% and 6.65%, while the FAR of the other five models mostly remained above 6.13%, with the maximum reaching 9.75%. The IBOA has obvious optimization effects, is not prone to becoming trapped in partial optima, and shows greatly improved accuracy.

## 7. Conclusions

Aiming at the difficulty of parameter optimization of wind turbine gearbox fault detection models, the IBOA-ERF fault detection model was proposed. The IBOA was used to optimize the hyperparameters of ERF, so as to improve the detection performance.

There are four main contributions of this paper: First, chaotic mapping is introduced to replace the original population initialization method to enhance the randomness of the population distribution and enhance the local development and global exploration capabilities. Second, the adaptive inertia weight factor is designed and combined with the landmark operator of PIO, so that the best position information of individual history is more effectively used, and it is integrated into the position update formula to improve the diversity and robustness of the BOA. Third, a new dynamic switching method of the search stage is designed, so that two search phases can reach a dynamic balance, preventing a drop into local optima and accelerating convergence. Finally, an improved fault detection model for wind turbine gearboxes is proposed by combining the above strategies with ERF.

In the experiments, MFO, MVO, SSA, SCA, BOA, and IBOA were introduced to enhance experimental fairness, each used to act on the ERF model, and the fitness function was constructed. MAR and FAR were used as assessment indicators. The results indicate that when using the IBOA to optimize the ERF parameters, the MAR and FAR are still low when the dataset is complex and the dimensionality is high.

Based on the proposed IBOA-ERF wind turbine gearbox fault detection model, the recommendations for future research are as follows:

- When the data categories are unbalanced—that is, when there are many normal samples and few fault samples—further research can be conducted to solve the problem of the model detection being biased towards the majority of samples, and the classification accuracy is reduced.
- With the upgrading of the wind turbine gearbox technology, the feature dimensionality and complexity of the original dataset can increase. There are many data pre-processing methods and no uniform measurement, which can influence the implementation of the model. The data pre-processing methods that are most suitable for this model can be further studied.
- The IBOA can be applied to other fault detection fields.

## Figures and Tables

**Table 1:** Basic test function information.

Function Types	Expressions	Scope	Optimal Value
Unimodal	F1(x) $=\overset{n}{\underset{i=1}{\sum }}x_{i}^{2}$	[−100,100]	0
	F2(x) $=\overset{n}{\underset{i=1}{\sum }}\left(\overset{n}{\underset{j=1}{\sum }}x_{j}^{2}\right)$	[−100,100]	0
	$F3\left(x\right)=\;\max\left\{\left|x_{i},1\leq i\ll n\right|\right\}$	[−100,100]	0
Multimodal	F4(x) $=\overset{n}{\underset{i=1}{\sum }}\left[x_{i}^{2}-10\cos\left(2{\pi x}_{i}\right)+10\right]$	[−5.12,5.12]	0
	F5(x) $=-20\exp\left(\sqrt{\frac{1}{n}\overset{n}{\underset{i=1}{\sum }}x_{i}^{2}}\right)-\exp\frac{1}{n}(\overset{n}{\underset{i=1}{\sum }}\left(\cos\left(2{\pi x}_{i}\right)\right)+20+e)$	[−32,32]	0
	F6(x) $=\frac{1}{4000}\overset{n}{\underset{i=1}{\sum }}x_{i}^{2}-\overset{n}{\underset{i=1}{\prod }}\cos\frac{x_{i}}{\sqrt{i}}+1$	[−600,600]	0

**Table 2:** Experimental results of the test functions.

Functions	Algorithms	Optimal Value	Worst Value	Average Value	Standard Deviation
F1	IBOA	**0**	**0**	**0**	**0**
	MFO	6.94 × 10^−1^	1.00 × 10^4^	1.35 × 10^3^	3.45 × 10^3^
	MVO	5.97 × 10^−1^	1.98 × 10^0^	1.26 × 10^0^	3.39 × 10^−1^
	SCA	6.02 × 10^−2^	3.30 × 10^2^	2.98 × 10^1^	6.89 × 10^1^
	SSA	3.05 × 10^−8^	3.04 × 10^−6^	2.70 × 10^−7^	5.77 × 10^−7^
	BOA	1.08 × 10^−11^	1.45 × 10^−11^	1.28 × 10^−11^	8.81 × 10^−13^
F2	IBOA	**0**	**0**	**0**	**0**
	MFO	8.24 × 10^−5^	6.67 × 10^3^	1.11 × 10^3^	2.29 × 10^3^
	MVO	2.44 × 10^−2^	2.48 × 10^−1^	1.07 × 10^−1^	5.59 × 10^−2^
	SCA	8.32 × 10^−9^	6.78 × 10^−2^	7.90 × 10^−3^	1.74 × 10^−2^
	SSA	3.09 × 10^−9^	1.91 × 10^−6^	1.50 × 10^−7^	3.78 × 10^−7^
	BOA	9.46 × 10^−12^	1.32 × 10^−11^	1.12 × 10^−11^	9.03 × 10^−13^
F3	IBOA	**0**	**0**	**0**	**0**
	MFO	2.93 × 10^−3^	1.04 × 10^1^	2.42 × 10^0^	3.35 × 10^0^
	MVO	3.34 × 10^−2^	1.98 × 10^−1^	9.98 × 10^−2^	4.08 × 10^−2^
	SCA	4.84 × 10^−7^	2.99 × 10^−2^	2.38 × 10^−3^	6.92 × 10^−3^
	SSA	1.47 × 10^−5^	1.02 × 10^−4^	2.82 × 10^−5^	1.94 × 10^−5^
	BOA	4.29 × 10^−9^	6.23 × 10^−9^	5.35 × 10^−9^	4.60 × 10^−10^
F4	IBOA	**0**	**0**	**0**	**0**
	MFO	8.95 × 10^0^	8.46 × 10^1^	2.47 × 10^1^	1.61 × 10^1^
	MVO	4.98 × 10^0^	3.38 × 10^1^	1.54 × 10^1^	7.15 × 10^0^
	SCA	0.00 × 10^0^	1.27 × 10^1^	6.42 × 10^−1^	2.58 × 10^0^
	SSA	3.98 × 10^0^	4.18 × 10^1^	1.80 × 10^1^	8.62 × 10^0^
	BOA	5.54 × 10^0^	5.61 × 10^1^	3.35 × 10^1^	1.94 × 10^1^
F5	IBOA	**8.88 × 10^−16^**	**8.88 × 10^−16^**	**8.88 × 10^−16^**	**0**
	MFO	1.13 × 10^0^	2.00 × 10^1^	1.11 × 10^1^	8.60 × 10^0^
	MVO	1.03 × 10^0^	3.36 × 10^0^	1.92 × 10^0^	4.99 × 10^−1^
	SCA	3.53 × 10^−2^	2.03 × 10^1^	1.11 × 10^1^	9.62 × 10^0^
	SSA	1.92 × 10^−1^	4.62 × 10^0^	2.65 × 10^0^	8.91 × 10^−1^
	BOA	4.49 × 10^−9^	6.87 × 10^−9^	6.01 × 10^−9^	5.29 × 10^−10^
F6	IBOA	**0**	**0**	**0**	**0**
	MFO	4.68 × 10^−2^	3.49 × 10^−1^	1.52 × 10^−1^	7.74 × 10^−2^
	MVO	1.35 × 10^−1^	5.77 × 10^−1^	3.32 × 10^−1^	1.20 × 10^−1^
	SCA	6.02 × 10^−13^	4.49 × 10^−1^	8.19 × 10^−2^	1.34 × 10^−1^
	SSA	6.64 × 10^−2^	6.44 × 10^−1^	2.46 × 10^−1^	1.49 × 10^−1^
	BOA	4.63 × 10^−14^	1.75 × 10^−11^	7.23 × 10^−13^	3.18 × 10^−12^

**Table 3:** Selection of parameters for optimization.

Parameter	Meaning	Value Range
n_estimators (τ)	The number of decision trees in ERF	[10, 1000]
max_depth (δ)	Maximum depth of the decision tree	[10, 200]

**Table 4:** Partial data of wind turbine operation.

Features	Time						
	18:12	18:13	18:14	….	19:40	19:41	19:42
nacelle_temperature	−8.5	−8.7	−8.8	….	9.3	9.5	9.8
wind_speed_1	10.01	9.94	9.34	….	5.92	6.12	6.01
….	….	….	….	….	….	….	….
hydraulic_main_sys_pressure	135.87	136.18	135.26	….	144.72	144.72	144.11
hydraulic_rotor_brake_sys_pressure	149.30	148.69	149.90	….	170.36	170.05	170.05

**Table 5:** Description of the datasets.

Dataset	Fault-Free	Faulty	Total Number of Samples	Total Number of Features
Dataset 1	1059	991	2050	210
Dataset 2	1211	1080	2291	210

**Table 6:** Confusion matrix for binary classification problems.

Actual Category	Predict Category	
	Normal	Fault
Normal	$S_{\mathrm{TN}}$ (true negative)	$S_{\mathrm{FP}}$ (false positive)
Fault	$S_{\mathrm{FN}}$ (false negative)	$S_{\mathrm{TP}}$ (true positive)

## Data Availability

The data that support the findings of this study are available from the corresponding author upon reasonable request.

## Nomenclature and Abbreviations

GWEC	Global Wind Energy Council
SCADA	Supervisory control and data acquisition
NFSW-BP-AdaBoost	Non-fuzzy solution-weighted BP-AdaBoost
XGBoost	Extreme gradient boosting
CS-GBDT	Cost-sensitive GBDT
RF	Random forest
ERF	Extreme random forest
BOA	Butterfly optimization algorithm
IBOA	Improved butterfly optimization algorithm
IBOA-ERF	Extreme random forest optimized by improved butterfly optimization algorithm
PIO	Pigeon-inspired optimization
MFO	Moth–flame optimization
MVO	Multi-verse optimization
SCA	Sine–cosine algorithm
SSA	Salp swarm algorithm
UCI	University of California Irvine
FAR	False alarm rate
MAR	Missing alarm rate
*Symbols*	
D	Number of numbers in ERF
$P_{t}(c|V_{i})$	The probability that the sample belongs to category c conditional on the feature vector $V_{i}$
$V_{i}$	Feature vector of the sample
$P(c|V_{i})$	Average value of $P_{t}(c|V_{i})$ in the ERF
c	Some kind of category
$\hat{c}$	Category corresponding to the maximum value of $P(c|f_{i})$
$I_{k}$	Mutual information of the two subsets
$H_{k}$	Split entropy of the feature k
$H_{c}$	Information entropy
${\mathrm{Score}}_{k}$	Score measurement of the calculated feature
f	Fragrance intensity
I	Stimulus intensity
s	Sensory modality
α	Power exponent
$x_{i}^{t}$	Location information of the i-th individual in the t-th iteration
$x_{i}^{t+1}$	Location information of the i-th individual in the t + 1-th iteration
r	Random value from 0 to 1
$g^{*}$	Best value in the current iteration
$f_{i}$	Fragrance intensity emitted by the i-th individual
j	Random number
k	Random number
X	Position parameter
t	Current number of iterations
μ	Logistics parameter
$T_{\mathrm{iter}}$	Largest value of the number of iterations t
ω	Adaptive inertia weight
e	Euler number
$x_{c}^{t}$	Position of the center of the flock in the current iteration
$\mathrm{Fit}\left(x_{i}^{t}\right)$	Value of the fitness function of the i-th individual
N	Number of individuals
∅	100*π
cov	Covariance
σ	Standard deviation
ρ	Correlation coefficient
S	Corresponding sample size
